# Hepatectomy outperforms microwave ablation for colorectal cancer liver metastases, with anatomical location affecting ablation outcomes

**DOI:** 10.3389/fsurg.2026.1878151

**Published:** 2026-07-20

**Authors:** Huan Yan, Huifang Xiong, Honghao Zhou, Cheng Zhou

**Affiliations:** 1 Department of Hepatobiliary Surgery, Wuhan No.1 Hospital (Wuhan Hospital of Traditional Chinese and Western Medicine), Wuhan, China; 2 Department of Digestive Medicine, People’s Hospital of Dongxihu District, Wuhan, China; 3 Department of Hepatic Surgery, Tongji Hospital, Tongji Medical College, Huazhong University of Science and Technology, Wuhan, China

**Keywords:** colorectal liver metastases, hepatectomy, high-risk lesions, incomplete ablation, liver resection, microwave ablation

## Abstract

**Objective:**

This study aimed to compare the clinical efficacy of hepatectomy and microwave ablation (MWA) for colorectal cancer liver metastases (CRLM), and to further clarify the clinical value and limitations of MWA in the treatment of high-risk CRLM lesions.

**Methods:**

A total of 98 patients with CRLM treated between July 2008 and February 2019 were retrospectively enrolled. Among them, 31 patients underwent hepatectomy and 67 received MWA. Tumor characteristics and long-term prognosis were compared between the two groups. A total of 105 CRLM lesions were further subgrouped into high-risk location (*n* = 36) and non-high-risk location (*n* = 69) for stratified analysis.

**Results:**

Patients treated with hepatectomy achieved significantly better recurrence-free survival (RFS) and overall survival (OS) than those treated with MWA. In the MWA cohort, lesions <3 cm in diameter could be completely ablated regardless of anatomical location. For tumors >3 cm, the complete ablation rate was significantly lower in high-risk lesions than in non-high-risk lesions. Multivariate analysis confirmed that tumor diameter was an independent risk factor for incomplete ablation.

**Conclusion:**

Hepatectomy provides superior long-term survival benefits over MWA for patients with CRLM. MWA exhibits unsatisfactory efficacy for high-risk lesions, especially those larger than 3 cm in diameter.

## Introduction

The liver is the most frequent site of distant metastasis in colorectal cancer (CRC). Approximately 50% of CRC patients develop colorectal liver metastases (CRLM) during the disease course (1), among whom 25% present with synchronous CRLM (sCRLM) at the initial diagnosis of the primary tumor (2). Progressive hepatic tumor burden remains the leading cause of mortality in CRC patients. Historically, CRLM was regarded as an end-stage malignant manifestation, and surgical hepatectomy was once considered to confer limited survival benefit (3, 4). Nevertheless, landmark research by De Haas et al. (5) confirmed that hepatectomy for CRLM does not increase perioperative mortality, but markedly improves long-term survival and elevates 5-year survival rates.

Currently, the general indications for curative hepatectomy in CRLM follow three core principles: radical resection of the primary colorectal lesion, complete removal of liver metastases with adequate residual hepatic functional reserve, and absence of unresectable extrahepatic metastatic lesions (6). Notably, tumor location, maximum diameter and lesion number are no longer absolute contraindications for hepatectomy. Both initially resectable lesions and initially unresectable lesions converted to resectable status via conversion therapy are recommended to receive timely surgical intervention. However, for high-risk location lesions—including those situated in deep hepatic regions or adjacent to major vascular structures such as the portal vein and inferior vena cava—conventional surgical resection is technically demanding and associated with increased perioperative risks. Accumulated evidence has revealed that simultaneous resection of primary CRC and synchronous liver metastases significantly elevates the risks of postoperative complications and short-term mortality (7).

As a minimally invasive local ablative technique, microwave ablation (MWA) exhibits prominent advantages including minimal trauma and reliable tumor eradication. For small hepatocellular carcinoma lesions <2 cm in diameter, MWA yields curative efficacy comparable to surgical resection (8, 9). Growing clinical evidence also demonstrates that combination therapy of MWA plus chemotherapy confers superior progression-free survival compared with chemotherapy monotherapy. Accordingly, MWA has emerged as a promising alternative for deep-seated, vessel-adjacent and surgically inaccessible CRLM lesions (10).

Nevertheless, the prognostic discrepancy between hepatectomy and MWA, as well as the clinical applicability and limitations of MWA for high-risk CRLM lesions, remain insufficiently clarified. Therefore, this retrospective study aimed to comprehensively compare the clinical efficacy and long-term survival outcomes of hepatectomy versus MWA in CRLM patients, and to further explore the efficacy of MWA in the management of high-risk location lesions, thereby providing clinical evidence for individualized treatment decision-making.

## Materials and methods

### Diagnosis of CRLM

This retrospective study enrolled 98 patients diagnosed with CRLM who received treatment at the Hepatic Surgery Center, Tongji Hospital, Tongji Medical College, Huazhong University of Science and Technology, between July 2008 and February 2019. All CRLM diagnoses were confirmed by pathological diagnosis or standardized imaging examinations, including contrast-enhanced ultrasonography (CEUS), contrast-enhanced computed tomography (CT), and contrast-enhanced magnetic resonance imaging (MRI). The cohort consisted of 66 males and 32 females, with ages ranging from 22 to 76 years and a mean age of 53 years.

### Patients

The inclusion criteria were as follows: (1) Histopathologically confirmed primary colorectal cancer combined with synchronous or metachronous liver metastases; (2) Curative resection of the primary colorectal tumor; (3) No more than three hepatic lesions with a maximum diameter <5 cm, or more than three lesions with a maximum diameter <3 cm; (4) Sufficient residual liver functional reserve after intervention; (5) Child–Pugh grade A liver function; (6) Normal coagulation function and platelet count >50 × 10⁹/L; (7) Absence of tumor thrombus in the portal vein or inferior vena cava.

All patients provided written informed consent before treatment, and the entire study protocol was conducted in accordance with the principles of the Declaration of Helsinki.

### Hepatectomy group

All hepatic resections were performed by experienced senior surgeons under general anesthesia. Open hepatectomy was conducted via a right subcostal oblique incision. For laparoscopic hepatectomy, a 5- or 6-port approach was adopted, and patients were placed in a head-up and feet-down position. Liver parenchymal dissection was performed using an ultrasonic scalpel, with large bile ducts and blood vessels routinely clipped for hemostasis and biliary tract control. Intraoperative ultrasonography was applied to precisely localize metastatic liver lesions and ensure complete resection.

### MWA subgroup

All patients in the MWA group received treatment in a supine position, with general or local anesthesia selected according to tumor size, depth and anatomical location. The ablation methods include percutaneous microwave ablation and direct-view ablation (including open abdominal microwave ablation and laparoscopic microwave ablation therapy). All microwave ablation procedures were completed under real-time ultrasound guidance. A 14-gauge water-cooled microwave antenna needle (effective length: 180 mm; ECO100A1, Nanjing ECO Medical Equipment Co., Ltd., Nanjing, China) was used for ablation. All operations were jointly performed by at least two qualified and experienced sonographers.

The microwave needle tip was placed 0.2–0.4 cm beyond the tumor base. The ablation power was set at 60 watts, with an ablation duration ranging from 4 to 10 min. Ablation was terminated when a continuous high-echoic area covering the entire tumor tissue persisted for at least 1 min under ultrasound monitoring, which indicated effective thermal coagulation corresponding pathologically to tumor necrosis. If the ablation range could not cover the entire tumor, multi-site ablation was performed, i.e., multiple ablations were applied to a single tumor until complete ablation was achieved. For lesions adjacent to major vessels or extrahepatic organs, microbubble changes around the electrode tip were closely monitored; ablation was terminated immediately once microbubbles approached vital structures.

For tumors adjacent to the gallbladder, stomach or diaphragm, an artificial ascites technique was applied for isolation and protection. Briefly, an 18-gauge puncture needle was inserted into the gap between the tumor and adjacent visceral organs under real-time ultrasound guidance, followed by slow infusion of 100 mL 0.9% normal saline. Continuous saline perfusion was maintained throughout the ablation process to reduce thermal injury to surrounding tissues. Single-needle or dual-needle puncture and multi-site ablation were performed individually based on tumor size and morphological characteristics.

### Definition of high-risk CRLM

Referring to previous preclinical and clinical studies (11–13), perivascular lesions were defined as tumors located within 5 mm of the first- or second-order branches of the portal vein or hepatic vein (axial diameter ≥ 3 mm) (14). Tumors within 5 mm of the heart, lung, gallbladder, right kidney or gastrointestinal tract were defined as lesions adjacent to extrahepatic organs (15).

All high-risk location nodules were evaluated strictly and independently. An abdominal radiologist with over 15 years of clinical experience reviewed and verified imaging findings. Two specialized sonographers further identified high-risk lesions by CEUS (EA720, ESAOTE S.p.A, Italy). The final judgment of high-risk nodules was determined by multidisciplinary consensus. Lesions sandwiched between multiple hepatic vascular structures or adjacent to multiple extrahepatic organs were also categorized into the high-risk location group, as these lesions were theoretically prone to incomplete ablation.

### Definition of complete ablation

Contrast-enhanced ultrasonography was routinely performed within 5 days after MWA to evaluate the immediate ablation effect. Incomplete ablation was defined as focal contrast filling in the target lesion during the arterial phase on CEUS, and supplementary microwave ablation was performed for confirmed residual tumors. In addition, enhanced CT or MRI within the first 3 months of follow-up showing new abnormal enhancement inside or at the margin of the ablation zone was also regarded as incomplete ablation. Complete ablation was defined as the absence of the above residual enhancement manifestations on postoperative imaging.

### Follow-up

All patients received regular postoperative imaging follow-up. CEUS, contrast-enhanced CT or MRI were arranged monthly within the first 6 months, and every 3 months thereafter. The follow-up deadline was October 30, 2020. Tumor recurrence was defined to the appearance of new lesions within the liver or the enlargement of previously ablated lesions. Patient survival information was collected by telephone interview and official mortality registration inquiry. The mean follow-up period was 40 months (median: 38 months; range: 4–94 months).

### Statistical analysis

Continuous variables were expressed as mean ± standard deviation, while categorical variables were presented as case numbers and percentages. Intergroup differences were compared using the *χ*^2^ test or independent Student's t-test as appropriate. Kaplan–Meier survival curves were plotted to analyze recurrence-free survival and overall survival, and intergroup differences were compared by log-rank test. A two-tailed *P* value <0.05 was considered statistically significant. All statistical analyses were performed using SPSS 19.0 software (IBM Corp., Armonk, NY, USA).

## Results

### Comparison of prognosis between hepatectomy group and MWA group

**Figure 1:**
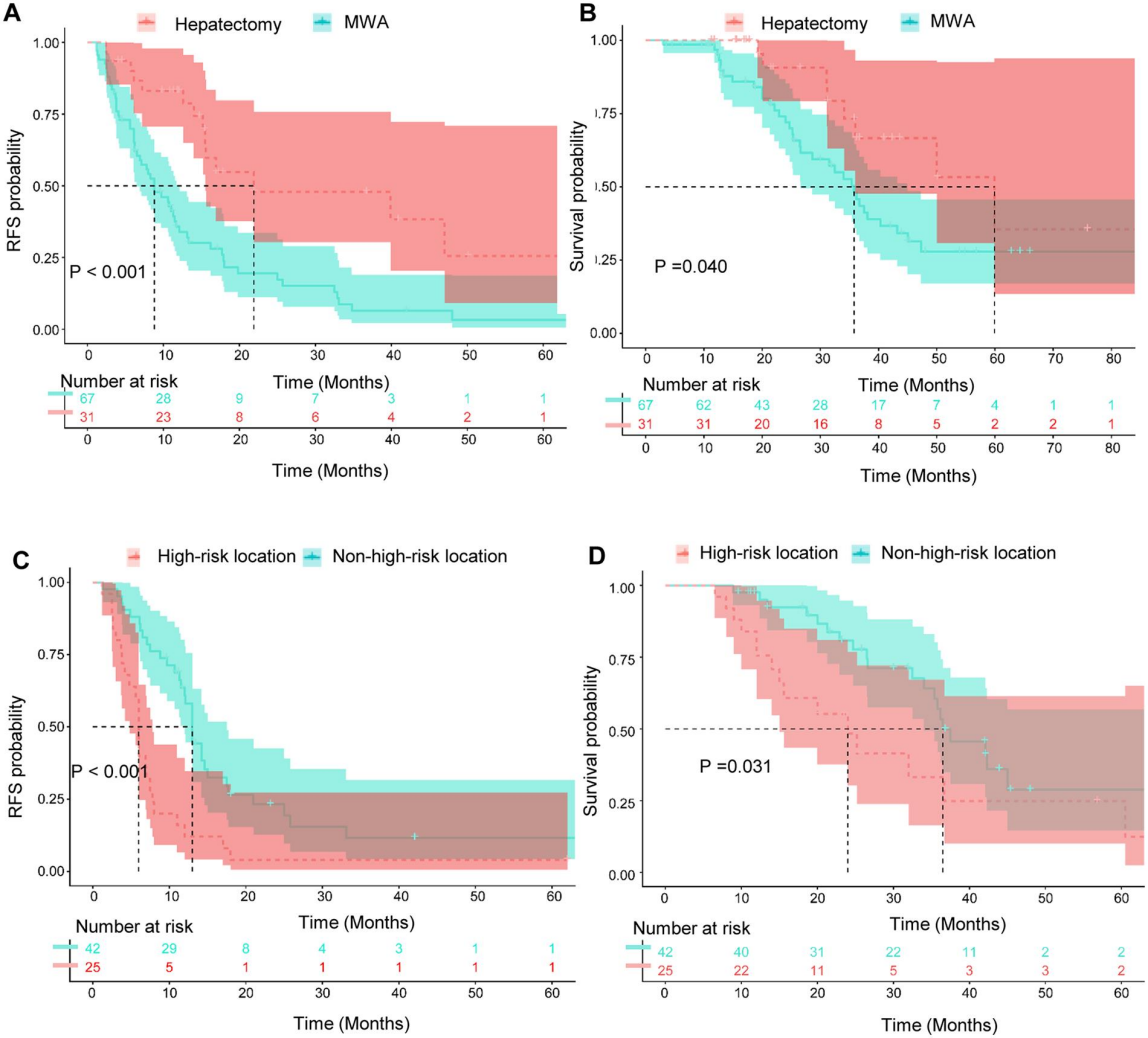
Kaplan–meier survival analysis of patients with colorectal cancer liver metastases (CRLM) according to treatment modality and tumor location. **(A)** Recurrence-free survival (RFS) curves comparing patients treated with microwave ablation (MWA, green) versus hepatectomy (red). The median RFS was significantly longer in the hepatectomy group than in the MWA group (*P* < 0.001, log-rank test). **(B)** Overall survival (OS) curves comparing the MWA and hepatectomy groups. Patients undergoing hepatectomy exhibited significantly better OS than those treated with MWA (*P* = 0.040, log-rank test). **(C)** Recurrence-free survival curves for patients in the MWA cohort stratified by tumor location (high-risk vs. non-high-risk). The high-risk location group had significantly shorter RFS than the non-high-risk group (*P* < 0.001, log-rank test). **(D)** Overall survival curves for patients in the MWA cohort stratified by tumor location. Patients with high-risk location lesions showed significantly poorer OS compared to those with non-high-risk lesions (*P* = 0.031, log-rank test).

Baseline clinical and pathological characteristics of patients in the hepatectomy group and MWA group are summarized in Table 1. No significant intergroup differences were detected in any demographic or tumor-related variables. The overall cohort was predominantly male, most patients presented with a solitary liver lesion, and more than half were diagnosed with synchronous CRLM. In the hepatectomy group, the recurrence rate was 45.2% (14/31) and the mortality rate was 25.8% (8/31). By contrast, the MWA group showed a markedly higher recurrence rate of 83.6% (56/67) and a mortality rate of 50.7% (34/67). Patients in the MWA group had significantly poorer recurrence-free survival (RFS) than those undergoing hepatectomy. The median RFS was 8.8 months [95% confidence interval (CI): 6.5–11.7 months] versus 21.9 months [95% CI: 15.5–not applicable (NA) months], with a significant difference (*P* < 0.001) (Figure 1A). Similarly, overall survival (OS) was significantly inferior in the MWA group, with a median OS of 35.8 months (95% CI: 26.6–45 months) compared with 59.9 months (95% CI: 36.0–NA months, *P* = 0.040) (Figure 1B).

**Table 1:** Baseline clinical characteristics of patients in the hepatectomy group and MWA group.

Clinical characteristics	Hepatectomy group (*n* = 31)	MWA group (*n* = 67)	*P* value
Age, years, median (IQR)	51 (45–58)	54 (47–63)	0.167
Gender, *n* (%)			0.684
Male	20 (64.5%)	46 (68.7%)	
Female	11 (35.5%)	21 (31.3%)	
BMI, kg/m^2^, median (IQR)	22 (21–25)	21 (20–23)	0.128
ECOG PS score, *n* (%)			0.303
0	31 (100%)	63 (94.0%)	
1	0 (0%)	4 (6.0%)	
Child-Pugh class, *n* (%)			1.000
A	31 (100%)	65 (97.0%)	
B	0 (0%)	2 (3.0%)	
Primary lesion location, *n* (%)			0.331
Left colon	16 (51.6%)	19 (28.4%)	
Right colon	6 (19.4%)	16 (23.9%)	
Rectum	9 (29.0%)	32 (47.7%)	
Tumor differentiation, *n* (%)			0.775
Well	3 (9.7%)	10 (14.9%)	
Moderate	22 (71.0%)	45 (67.2%)	
Poor	6 (19.3%)	12 (17.9%)	
T stage, *n* (%)			0.838
T1	1 (3.2%)	2 (3.0%)	
T2	11 (35.5%)	28 (41.8%)	
T3	19 (61.3%)	37 (55.2%)	
Lymph node metastasis, *n* (%)			0.143
N0	17 (54.8%)	32 (47.8%)	
N1	5 (16.1%)	23 (34.3%)	
N2	9 (29.0%)	12 (17.9%)	
Preoperative CEA, ng/mL, median (IQR)	48 (2.6–128)	57 (11.4–128)	0.828
Liver metastasis interval, *n* (%)			0.881
> 12 months	12 (38.7%)	27 (40.3%)	
≤ 12 months	19 (61.3%)	40 (59.7%)	
Number of liver lesions, *n* (%)			0.468
Single	18 (58.1%)	44 (65.7%)	
Multiple	13 (41.9%)	23 (34.3%)	
Maximum lesion diameter, cm, median (IQR)	3.0 (1.5–5.0)	2.7 (1.3–5.0)	0.051
Timing of liver metastasis, *n* (%)			0.294
Synchronous	15 (48.4%)	40 (59.7%)	
Metachronous	16 (51.6%)	27 (40.3%)	
Neoadjuvant therapy, *n* (%)			0.845
Yes	20	44	
No	11	23	
Postoperative adjuvant therapy, *n* (%)			0.832
Yes	29	62	
No	2	5	

BMI, body mass index; ECOG PS, Eastern Cooperative Oncology Group performance status; CEA, carcinoembryonic antigen; MWA, microwave ablation; CRLM, colorectal liver metastases; CRS, Clinical Risk Score.

T stage definitions: T1, tumor invades submucosa; T2, tumor invades muscularis propria; T3, tumor penetrates muscularis propria into pericolorectal tissues.

N stage definitions: N0, no regional lymph node metastasis; N1, metastasis in 1–3 regional lymph nodes; N2, metastasis in 4 or more regional lymph nodes.

### Comparison of prognosis between different locations

We further compared prognostic outcomes between high-risk and non-high-risk location lesions in the MWA cohort. The high-risk location group demonstrated significantly shorter RFS than the non-high-risk group, with a median RFS of 6.0 months (95% CI: 4.8–8.9 months) versus 12.4 months (95% CI: 10–15.6 months, *P* < 0.001) (Figure 1C). Consistently, lesions in high-risk locations were associated with shorter OS than those in non-high-risk locations; the median OS was 25.0 months (95% CI: 15.6–NA months) versus 37.8 months (95% CI: 36.2–NA months, *P* = 0.031) (Figure 1D).

### Nodule location and ablation efficacy

A total of 105 nodules were included in this analysis, and the detailed lesion distribution according to high-risk anatomical location is summarized in Table 2. Thirty-six nodules were categorized into the high-risk location group and 69 into the non-high-risk location group. The incomplete ablation rate was 33.3% (12/36) in the high-risk group versus 15.9% (11/69) in the non-high-risk group, with a significant intergroup difference (*χ*^2^ = 4.147, *P* = 0.042). Subgroup analysis by tumor diameter showed that 16 (44.4%) of 36 high-risk nodules and 21 (30.4%) of 69 non-high-risk nodules had a diameter exceeding 3 cm. For tumors <3 cm in diameter, no significant difference was observed in the complete ablation rate between the two groups (17/20 vs. 42/48, *χ*^2^ = 0.078, *P* = 0.780). However, for tumors >3 cm, the high-risk location group had a markedly lower complete ablation rate than the non-high-risk group (7/16 vs. 16/21, *χ*^2^ = 4.063, *P* = 0.044).

**Table 2:** Distribution of 36 high-risk location nodules.

Perivascular locations	*n* = 17	Adjacent to extrahepatic organs	*n* = 19
Right portal vein trunk	2	Gallbladder	5
Left portal vein trunk	0	Right kidney	0
Anterior branch of right portal vein	4	Stomach	0
Posterior branch of right portal vein	3	Colon	0
Middle hepatic vein trunk	2	Heart	1
Right hepatic vein trunk	3	Diaphragm	13
Inferior vena cava	3		

High-risk locations were defined as nodules adjacent to major vessels (perivascular) or nodules within 1 cm of extrahepatic organs.

Univariate logistic regression analysis identified poor tumor differentiation, tumor diameter >3 cm, multiple tumors, high-risk location, CEA >200 ng/mL, synchronous liver metastasis, absence of preoperative neoadjuvant therapy, and lack of postoperative adjuvant treatment as risk factors for incomplete ablation. Multivariate logistic regression analysis further confirmed that tumor diameter >3 cm was the only independent risk factor for incomplete ablation [odds ratio (OR) = 7.545, 95% CI: 3.544–10.548, *P* = 0.025] (Table 3).

**Table 3:** Univariate and multivariate logistic regression analyses for incomplete ablation.

Variables	Univariate analysis	*P* value	Multivariate analysis	*P* value
	OR (95% CI)		OR (95% CI)	
Gender (male vs. female)	1.054 (0.854–1.132)	0.185	NA	NA
Age (<50 vs. ≥ 50 years)	0.954 (0.832–1.358)	0.241	NA	NA
Primary tumor differentiation (poor vs. well/moderate)	1.745 (1.521–3.254)	0.012	1.951 (0.845–6.654)	0.542
Tumor diameter (>3 vs. ≤ 3 cm)	3.524 (1.277–31.755)	< 0.001	7.545 (3.544–10.548)	0.025
Tumor number (multiple vs. single)	1.257 (1.127–2.745)	0.034	1.587 (0.954–3.698)	0.367
Artificial ascites (yes vs. no)	0.955 (0.914–1.237)	0.254	NA	NA
Tumor location (high-risk vs. non-high-risk)	3.284 (2.157–7.561)	0.014	5.254 (0.714–9.654)	0.124
Initial CEA (≥200 vs. < 200 ng/mL)	1.257 (1.047–3.274)	0.029	3.258 (0.952–6.954)	0.375
Liver metastasis interval (>12 vs. ≤ 12 months)	0.982 (0.851–1.587)	0.247	NA	NA
Liver metastasis timing (synchronous vs. metachronous)	1.878 (1.074–4.544)	0.017	2.665 (0.785–10.652)	0.521
Neoadjuvant therapy (no vs. yes)	1.905 (1.835–3.985)	0.008	2.041 (0.851–5.542)	0.686
Postoperative adjuvant therapy (no vs. yes)	3.258 (1.852–12.639)	0.025	5.154 (0.635–17.554)	0.415

CEA, carcinoembryonic antigen; OR, odds ratio; CI, confidence interval; NA, not applicable (variables not included in the multivariate model due to non-significance in univariate analysis).

**Figure 2:**
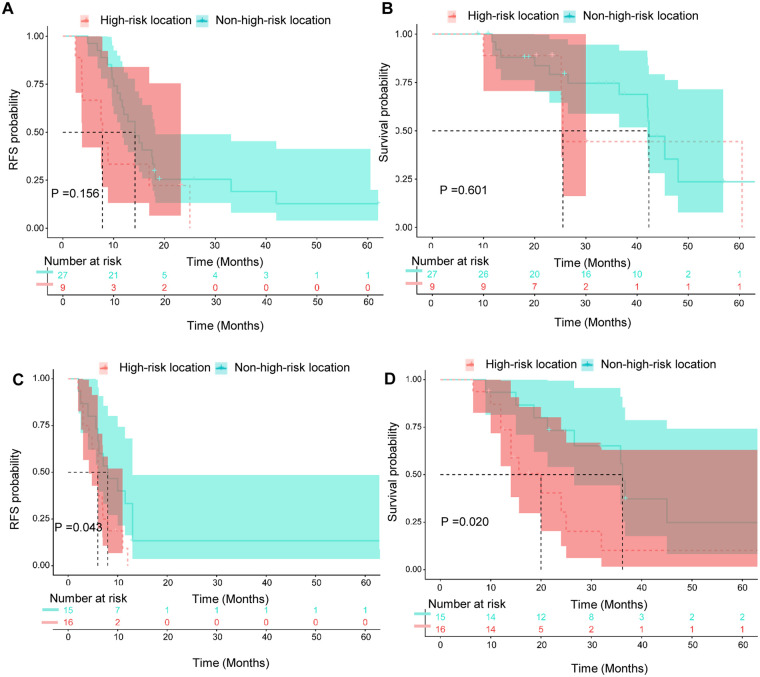
Kaplan–meier survival analysis stratified by tumor diameter and anatomical location in patients with colorectal cancer liver metastases treated with microwave ablation. **(A)** Recurrence-free survival (RFS) curves for patients with tumors <3 cm in diameter, comparing lesions in high-risk locations (red) versus non-high-risk locations (cyan). No significant difference in RFS was observed between the two groups (*P* = 0.156, log-rank test). **(B)** Overall survival (OS) curves for patients with tumors <3 cm in diameter, stratified by anatomical location. There was no significant difference in OS between the high-risk and non-high-risk location groups (*P* = 0.601, log-rank test). **(C)** Recurrence-free survival curves for patients with tumors >3 cm in diameter, comparing high-risk versus non-high-risk location lesions. Patients with high-risk location tumors had significantly shorter RFS than those with non-high-risk location tumors (*P* = 0.043, log-rank test). **(D)** Overall survival curves for patients with tumors >3 cm in diameter, stratified by anatomical location. Patients with high-risk location lesions exhibited significantly poorer OS compared to those with non-high-risk location lesions (*P* = 0.020, log-rank test).

We further compared ablation efficacy and survival outcomes stratified by tumor location within different diameter subgroups. In the subgroup of tumors <3 cm, no significant differences were found between the high-risk and non-high-risk location groups in terms of RFS [7.8 months (95% CI: 3.8–NA months) vs. 14.2 months (95% CI: 11.7–18.2 months), *P* = 0.156] (Figure 2A) or OS [25.5 months (95% CI: 25.2–NA months) vs. 42.3 months (95% CI: 42.0–NA months), *P* = 0.601] (Figure 2B). For tumors >3 cm, patients in the high-risk location group had significantly inferior RFS [6.0 months (95% CI: 4.2–NA months) vs. 8.0 months (95% CI: 6.0–NA months), *P* = 0.043] (Figure 2C) and OS [20 months (95% CI: 14.0–NA months) vs. 36.2 months (95% CI: 26.6–NA months), *P* = 0.020] compared with those in the non-high-risk location group (Figure 2D).

### Cases

**Figure 3:**
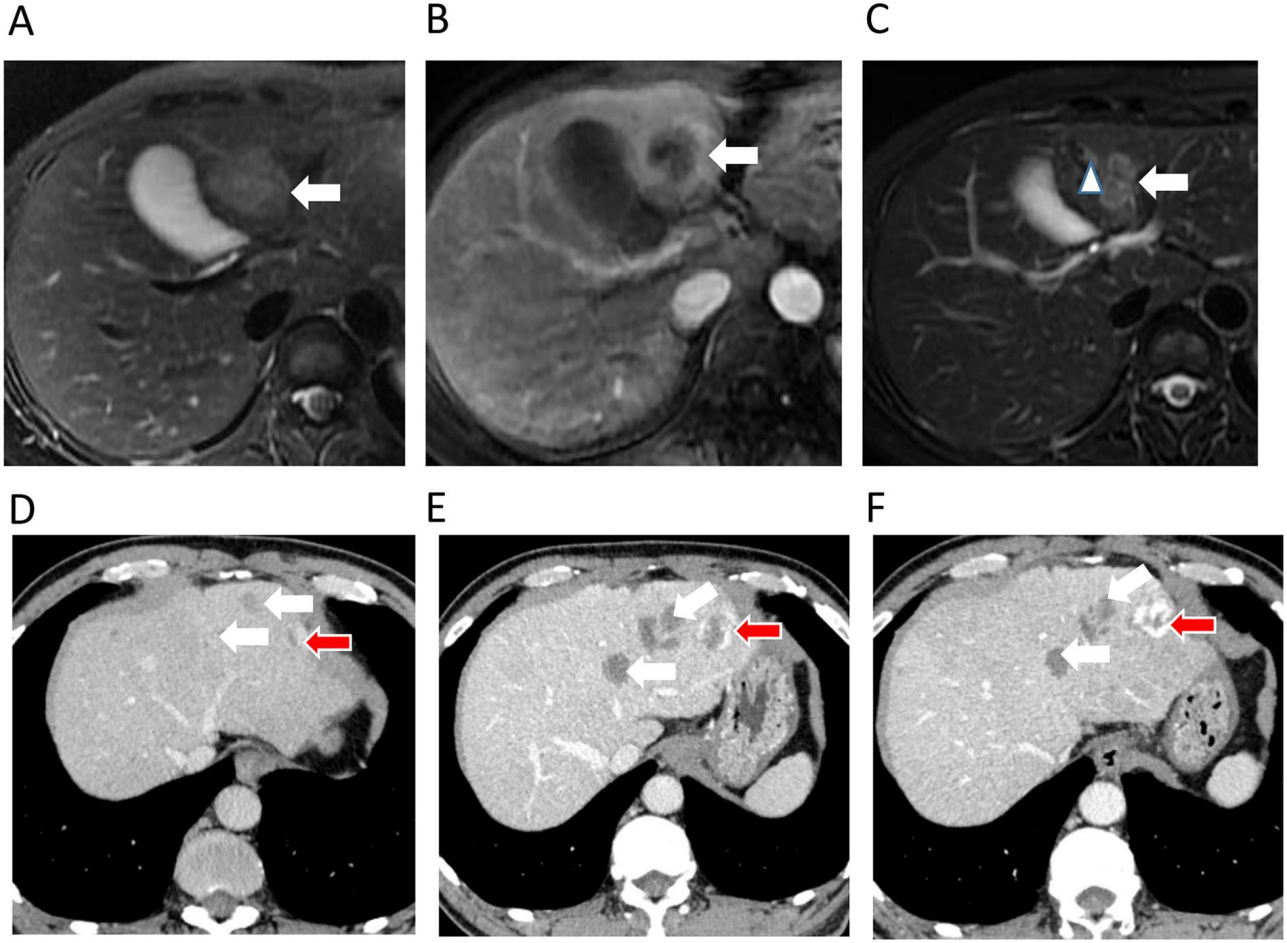
Representative imaging findings of two patients with high-risk location colorectal cancer liver metastases (CRLM) who developed incomplete ablation or local progression following microwave ablation (MWA). Panels A–C depict serial MRI scans from a 42-year-old male patient with a high-risk CRLM lesion adjacent to the gallbladder: **(A)** Baseline pre-treatment MRI, showing the index lesion (white arrow) located adjacent to the gallbladder, a high-risk anatomical location. **(B)** MRI at 3 months post-MWA, demonstrating persistent enhancement at the ablation site (white arrow), consistent with viable residual tumor. **(C)** MRI at 6 months post-MWA, confirming ongoing viable tumor at the primary ablation site (white arrow) with the development of a new satellite lesion (white triangular arrow) adjacent to the primary tumor. Panels D–F show serial contrast-enhanced computed tomography (CT) scans from a 58-year-old male patient with multiple CRLM lesions, including a high-risk lesion adjacent to the diaphragm: **(D)** Baseline pre-treatment CT, identifying multiple index lesions: one high-risk lesion adjacent to the diaphragm (red arrow) and additional liver metastases (white arrows). **(E)** CT at 3 months post-MWA, revealing abnormal enhancement surrounding the diaphragmatic lesion (red arrow), suggestive of residual tumor activity at the ablation margin. **(F)** CT at 6 months post-MWA, demonstrating persistent and increased enhancement at the diaphragmatic lesion (red arrow), confirming local progression of the high-risk CRLM lesion. All arrows indicate the locations of the target lesions. These two cases exemplify suboptimal treatment outcomes, including incomplete ablation and local recurrence, in high-risk location CRLM treated with MWA.

Figure 3 illustrates the process of MWA treatment for a 42-year-old male patient. T2-weighted MRI of the upper abdomen showed a CRLM lesion located beside the gallbladder (indicated by the white arrow, Figure 3A). Three months after ablation, enhanced MRI of the upper abdomen during the arterial phase showed enhancement at the tumor margin (indicated by the white arrow, Figure 3B). Six months after ablation, re-examination T2-weighted MRI of the upper abdomen showed residual tumor activity (indicated by the white arrow) accompanied by suspicious metastatic foci around the lesion (indicated by the white triangular arrow, Figure 3C).

Figure 3D shows the process of MWA treatment for another 58-year-old male patient. Enhanced CT of the upper abdomen during the venous phase showed three lesions (indicated by red and white arrows) in the left medial and left lateral lobes of the liver, one of which was adjacent to the left diaphragm. Three months after ablation, enhanced CT of the upper abdomen during the venous phase showed incomplete ablation and residual tumor activity (indicated by the red arrow, Figure 3E). Six months later, enhanced CT of the upper abdomen during the venous phase showed tumor growth and more pronounced enhancement compared with baseline (indicated by the red arrow, Figure 3F).

## Discussion

Current clinical consensus on the management of CRLM holds that surgical intervention should be performed promptly for initially resectable lesions to achieve R0 resection, as well as for initially unresectable lesions that become resectable following conversion therapy (16–18). Surgery remains the most effective curative modality for CRLM. This retrospective study analyzed the short-term and long-term outcomes of 31 patients who underwent hepatectomy and 67 patients who received MWA, and the results clearly demonstrated that hepatectomy is superior to MWA in terms of disease control and long-term survival. Previous studies have suggested that patients with CRLM may benefit from multiple surgical interventions if their general condition permits (19). However, factors such as insufficient residual liver volume and the increasing technical complexity of repeated hepatectomies limit the feasibility of multiple surgical procedures in most patients with frequent intrahepatic recurrence. In the present study, only 4 out of 31 patients who underwent hepatectomy received multiple liver resections; the main obstacles included long-term chemotherapy-related toxicity, poor physical status, and inadequate residual liver function, all of which compromised the feasibility of additional surgical resections. Furthermore, synchronous resection of the primary colorectal tumor and liver metastases is associated with prolonged operative time and increased intraoperative blood loss, which elevate perioperative risks. Given these challenges, the application of MWA for intrahepatic lesions during surgery—especially for deep-seated hepatic lesions—represents a more rational treatment strategy. Although the efficacy of hepatectomy is significantly superior to that of MWA alone, combination therapy with MWA and postoperative chemotherapy yields better outcomes than chemotherapy monotherapy (20). Therefore, the judicious use of MWA combined with postoperative chemotherapy may improve the long-term survival prospects of eligible patients. Various thermal and non-thermal ablation modalities have been developed for the treatment of hepatocellular carcinoma, each with distinct advantages and limitations. RFA, the most widely established technique, utilizes high-frequency alternating current to generate frictional heat within tumor tissue. While RFA offers excellent local control for small (<3 cm) tumors, it is limited by the “heat sink effect” adjacent to large blood vessels, longer ablation times, and higher risk of skin burns (21, 22). MWA, the technique employed in the present study, overcomes several limitations of RFA. MWA achieves higher intratumoral temperatures (up to 120 °C) more rapidly, creates larger and more predictable ablation zones, and is less affected by the heat sink effect, making it particularly advantageous for tumors near vascular structures or larger nodules (3–5 cm) (23). Additionally, MWA allows simultaneous multi-antenna ablation with synergistic heating, further improving treatment efficiency. Recent meta-analyses have demonstrated comparable or superior oncological outcomes of MWA versus RFA for early-stage HCC, with reduced procedure time and lower major complication rates (24). MWA represents a favorable balance of efficacy, safety, and practicality for HCC ablation, with particular advantages in vascular proximity and treatment speed. The choice of ablation modality should be individualized based on tumor size, location, proximity to vital structures, and institutional expertise. It is worth noting that MWA has several inherent technical limitations. The most significant issue lies in the difficulty of accurately predicting the size and shape of the thermal damage area, and the operation is highly dependent on experience. There is a significant difference in results between experienced interventional radiologists and those without experience. These technical limitations collectively lead to a higher local tumor progression rate after MWA compared to edge-negative surgical resection.

A key factor contributing to the inferior efficacy of MWA compared with hepatectomy is the high incidence of incomplete ablation. Clinically, during the follow-up of patients treated with MWA, it is occasional to observe enlargement or peripheral spread of previously ablated hepatic lesions. Additionally, lesions located near the hepatic capsule may rupture following MWA, leading to intra-abdominal implantation metastasis. Current research generally consensus that ablation therapy is most suitable for primary or metastatic hepatic tumors with a diameter of less than 3 cm (25–27). In practice, the ablation area generated by microwave energy is approximately equivalent to a sphere with a diameter of 3 cm; thus, achieving complete ablation becomes increasingly challenging as tumor diameter exceeds 3 cm. Moreover, lesion location is a critical factor influencing the success of complete ablation. Currently, there remains ongoing debate regarding the safety and efficacy of ablation for lesions in high-risk locations (36). Some studies have reported no significant differences in complete ablation rates or complication rates between high-risk and non-high-risk locations, noting that perivascular lesions can still achieve complete tumor necrosis through ablation, and MWA can be performed adjacent to extrahepatic organs (e.g., gallbladder, colon) without causing damage to these structures (28–30). However, other researchers argue that during the ablation of perivascular lesions, the “heat sink effect”—whereby blood flow dissipates thermal energy—prevents the tumor tissue adjacent to blood vessels from reaching the temperature required for complete necrosis (31). Furthermore, although MWA is considered a minimally invasive local treatment, it carries potential risks such as hollow organ perforation and diaphragmatic injury when targeting lesions adjacent to extrahepatic organs (32, 33). Additionally, when balancing the goal of complete ablation with the need to avoid severe complications, clinicians often prioritize complication prevention, leading to premature termination of the ablation process before the entire tumor area is adequately treated. This shortened ablation duration increases the risk of incomplete ablation, allowing residual tumor cells to proliferate and form visible lesions in a short period. In the present study, variables (including tumor size, tumor location, and CEA levels) were analyzed, and the results indicated that tumor diameter is an independent risk factor for incomplete ablation. Larger tumors are more likely to be located adjacent to blood vessels and extrahepatic organs; consequently, under the combined influence of these two factors, ablation of such lesions is often incomplete and may even lead to local tumor progression.

This study demonstrated that patients with nodules in high-risk location exhibited significantly poorer prognosis than those with nodules in non-high-risk location. Further subgroup stratification revealed that for patients with tumors larger than 3 cm, nodules located in high-risk areas carried an elevated risk of incomplete ablation, and the tumors showed rapid progression. By contrast, for patients with tumors smaller than 3 cm, complete ablation was achievable regardless of whether the nodules were situated in high-risk or non-high-risk location. This study indicates that tumor location in high-risk areas constitutes the fundamental underlying cause for the failure to achieve complete ablation in nodules larger than 3 cm.

This study has several limitations. First, the relatively small sample size and single-center design may limit the generalizability of the results, highlighting the need for prospective, multicenter, randomized controlled trials to validate our findings. Second, the definition of perivascular tumors remains a matter of debate, and no globally standardized diagnostic criteria have been established for complete ablation (34, 35). Third, as a retrospective study, it is susceptible to potential confounding factors and selection bias; for instance, interpatient variations in the dosage and duration of postoperative adjuvant chemotherapy may have affected the treatment outcomes. Additionally, this study did not account for differences in prognosis or therapeutic strategies among patients with mutations in key genes such as MMR, MSI, KRAS, NRAS, and BRAF. Furthermore, the long enrollment period may have introduced substantial heterogeneity in the regimens of neoadjuvant and postoperative adjuvant treatments.

## Data Availability

The original contributions presented in the study are included in the article/Supplementary Material, further inquiries can be directed to the corresponding author.
